# Influence of Internet-Based Health Management on Control of Blood Glucose in Patients with Type 2 Diabetes: A Four-Year Longitudinal Study

**DOI:** 10.3390/healthcare13050553

**Published:** 2025-03-04

**Authors:** Yuyang Wang, Qiang Hu, Botian Chen, Lingfeng Dai, Chun Chang, Defu Ma

**Affiliations:** School of Public Health, Peking University Health Science Center, 38 Xueyuan Road, Haidian District, Beijing 100191, China; wyuyang2018@163.com (Y.W.); qianghu@stu.pku.edu.cn (Q.H.); cbt970812@163.com (B.C.); 1910301338@pku.edu.cn (L.D.);

**Keywords:** diabetes, internet-based health management, blood glucose control, health behavior, self-management

## Abstract

**Background:** Diabetes is a major chronic disorder that significantly impacts life expectancy and imposes substantial economic burdens on individuals and healthcare systems. Internet-based health management has emerged as an innovative approach to support diabetes care by facilitating sustainable behavioral change and improving health outcomes. **Objective:** This longitudinal study aims to evaluate the impact of internet-based health management on blood glucose control in type 2 diabetes (T2D) patients over four years. **Methods:** A total of 30,333 participants were recruited from five provinces in China in 2013, including 2307 T2D patients. Participants utilized a comprehensive internet-based health management platform that provided personalized diet plans, exercise recommendations, and psychological support. Data were collected through regular health examinations and questionnaires, and logistic regression was conducted to identify key factors associated with effective blood glucose control. **Results:** After four years, the diabetes awareness rate among T2D patients increased from 17.72% to 19.84%, and the control rate rose from 7.22% to 26.91%. Notable improvements were observed in health-related behaviors, including smoking cessation, increased physical activity, and healthier dietary habits, particularly in the consumption of vegetables, fruits, soybeans, and nuts. Clinical outcomes also showed significant improvement, with reductions in fasting blood glucose (FBG), total cholesterol (TC), triglycerides (TG), and low-density lipoprotein cholesterol (LDL-C). Key factors contributing to effective blood glucose control in T2D patients included smoking cessation, increased intake of soybeans, nuts, and fruits, and reduced sedentary time. **Conclusions:** Internet-based health management significantly improved blood glucose control and health behaviors in T2D patients. This study confirms the potential of internet-based health management strategies to overcome geographical and healthcare access barriers, providing evidence for diabetes care in underdeveloped regions.

## 1. Introduction

Diabetes is one of the most widespread chronic diseases globally, presenting significant challenges to both individual health and healthcare systems. It not only reduces life expectancy but also imposes substantial economic burdens on patients and society as a whole [1,2]. Recent estimates indicate that the global prevalence of diabetes reached 8.63% in 2017, and this figure continues to rise [3,4]. In China, the prevalence is even higher, with 12.56% of adults over the age of 45 diagnosed with diabetes in the same year [5,6,7]. As this trend persists, diabetes will remain a critical public health challenge in the coming decades [8].

Type 2 diabetes (T2D) is associated with severe complications, including diabetic foot, hypoglycemia, and retinopathy, all of which significantly affect patients’ quality of life [9]. Despite the fact that there is currently no cure for this chronic disease, effective blood sugar control has been proven to reduce the incidence of complications and improve the quality of life for patients with diabetes [10,11]. Current clinical treatment options for T2D include oral hypoglycemic agents, insulin therapy, and glucagon-like peptide-1 (GLP-1) receptor agonists. Drug therapy regulates blood sugar levels to control the progression of the disease, but its effectiveness is often limited by patient adherence and potential side effects, which are insufficient to achieve optimal disease management [12]. Lifestyle changes, such as adjusting dietary structure, increasing physical activity, and maintaining a healthy weight, have been proven to be crucial components of T2D management, accounting for over 50% of the effectiveness in diabetes prevention and control [13,14].

Nevertheless, achieving optimal diabetes management remains a significant challenge. A large-scale, nationally representative cross-sectional survey conducted in China in 2013, involving 170,287 participants, revealed that only 37% of T2D patients were aware of their diagnosis, and merely 32% were receiving treatment [15]. This underscores a critical gap in awareness and management, suggesting that many T2D patients lack the knowledge and resources necessary for effective disease management.

Health management, which focuses on modifying unhealthy behaviors and mitigating risk factors, has emerged as a promising strategy for chronic disease control [16,17]. Effective health management fosters sustainable behavioral change, enabling T2D patients to gain knowledge, boost self-efficacy, and better manage their diabetes over the long term [18,19]. The provision of adequate information and consistent support plays a crucial role in empowering patients to manage chronic diseases.

Conventional diabetes management generally necessitates regular face-to-face consultations between patients and healthcare providers [20]. As a result, patients’ health management often relies on infrequent visits and irregular follow-ups, which leads to suboptimal control of diabetes [21,22]. With the rising prevalence of diabetes, limited healthcare resources are struggling to meet the growing demand for long-term, continuous management of the disease.

Internet-based health management, as an emerging form of remote management, has been proven to be both feasible and effective for diabetes care [23,24]. A randomized controlled trial conducted among Asians showed that, compared to conventional care, internet-based lifestyle interventions were more effective in reducing body weight and blood glucose levels in adults with T2D [25,26]. Another study, which used email, online platforms, and mobile phones for lifestyle interventions in patients with prediabetes, demonstrated that mobile health platforms had a significant positive impact on blood glucose control, weight, waist circumference, and diabetes risk [27]. The application of internet-based health management also faces some challenges. For instance, issues such as the digital divide, insufficient internet coverage, and patients’ technical familiarity may limit its effectiveness in specific populations. In the western regions of China, where the prevalence of diabetes is rising, the effectiveness of internet-based health management remains unclear.

Therefore, this study aims to explore the effectiveness of internet-based health management on blood glucose control and health-related behaviors in T2D patients and analyze the key factors influencing the effectiveness of blood glucose control, offering potential insights into cost-effective methods for diabetes management in developing countries and regions.

## 2. Methods

### 2.1. Study Design

This study adopted a longitudinal design to evaluate the effectiveness of internet-based health management in improving the management of diabetes in patients. A longitudinal approach was chosen to capture the dynamic changes in glycemic control and health behavior changes (e.g., dietary habits) over time, enabling the evaluation of the intervention’s sustained effects. Recruitment took place from January to May 2013, with initial health assessments and participant enrollment finalized in the same year. The follow-up period began in July 2017 and ended in October 2017, resulting in a 4-year interval selected based on the natural progression of T2D and the need to observe long-term health outcomes. All participants signed informed consent prior to the study and were able to understand the study’s purpose and procedures. This study adhered to the guidelines of the Declaration of Helsinki (1975) and was approved by the Biomedical Ethics Committee of Peking University (IRB00001052-0816) [28].

### 2.2. Participants

The inclusion criteria for this study were as follows: (1) participants should be 18 years of age or older to ensure accurate assessment of intervention effects; (2) participants should be employees taking part in the corporate health management program, who have access to health management services provided by the company, facilitating both intervention implementation and data collection; (3) participants should possess the ability to use a smartphone and access the internet, meeting the technical requirements for internet-based health management interventions; (4) participants should be willing to download and regularly use the health management mobile application, ensuring the sustained effectiveness of the intervention measures; (5) there should be an absence of severe cognitive impairment or major psychiatric disorders to avoid impacting participants’ understanding and execution of health management requirements.

Exclusion criteria were as follows: (1) individuals with severe diseases such as end-stage renal disease and severe heart disease due to their complex medical needs; (2) pregnant women or those planning to become pregnant, whose health management strategies primarily focus on prenatal care; (3) individuals planning to relocate or unable to be present for long-term follow-up, as continuous follow-up is crucial for assessing the long-term effects of the intervention and avoiding significant data loss due to these circumstances.

Participants were recruited voluntarily from the employees of the petroleum enterprise in the five provinces. The recruitment process included information sessions about the study, demonstrations of the internet health platform, and a detailed review of the screening criteria. Volunteers were recruited from the enterprise’s branch offices, and all participants signed informed consent before enrollment. The recruitment process included both face-to-face communication and appropriate digital methods for supplementation.

### 2.3. Setting

**Figure 1:**
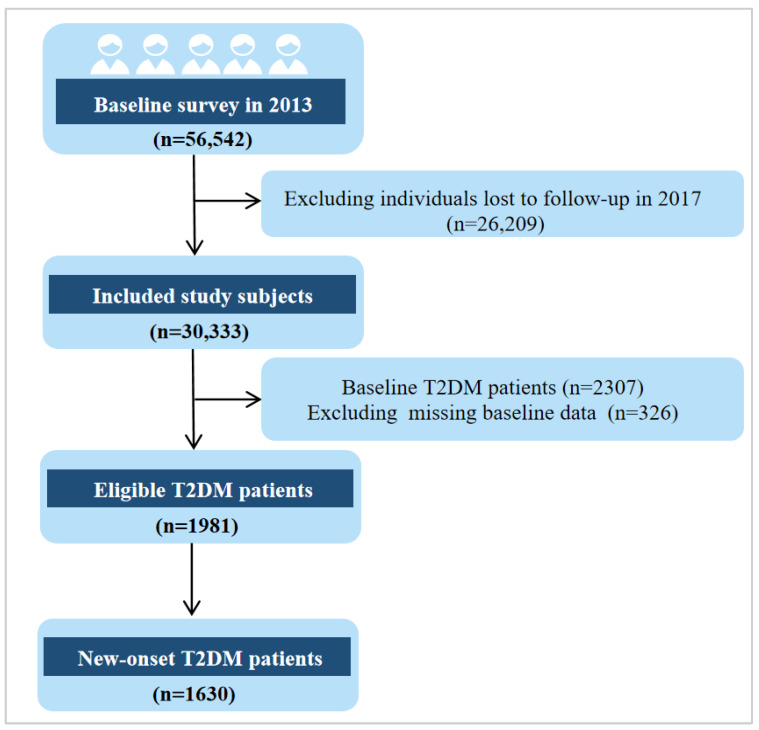
Flowchart of T2DM patient selection from the 2013 baseline survey.

At baseline, a total of 56,542 participants underwent physical examinations and biochemical tests, which were conducted at local community health service centers. These assessments included physical examinations, blood biochemical tests, and completion of detailed questionnaires, which collected basic demographic information, lifestyle factors, and dietary habits. The follow-up period began in July 2017, four years after the baseline survey. During the follow-up, 30,333 participants completed a second round of physical examinations and follow-up questionnaires. Figure 1 showed the process of selecting T2D study subjects from the 2013 baseline survey.

### 2.4. Self-Health Management Platform

An employee health management platform was developed for the staff of China Changqing Petroleum Corporation. The platform is organized into three key areas: “Know My Health”, “Improve My Health”, and “My Health Management Outcomes”. Each participant could log in by entering their personal password.

In the first section, “Know My Health”, participants could access their personal health records, which included health examination results and questionnaire assessments. The second section, “Improve My Health”, offered personalized health management plans tailored to individual health conditions. These plans included recommendations for diet, exercise, psychological well-being, and other behavioral changes aimed at mitigating existing health risks. The health management plans were developed based on assessments from healthcare professionals specializing in clinical nutrition and exercise, combined with expert group discussions to ensure a comprehensive approach to health management.

In the third section, “My Health Management Outcomes”, participants could reassess their health status after three months of following their personalized health management plan. They could review their progress and receive feedback. Based on these results, the platform adjusted subsequent management plans to optimize health outcomes. The health records included basic demographic information (e.g., age, gender, height, weight), clinical parameters from health examinations (e.g., blood pressure, blood glucose levels, blood lipid levels), personal and family medical histories, and lifestyle factors such as dietary and exercise habits.

Throughout the process of internet-based health management, healthcare professionals provided remote, personalized support, primarily through phone calls or text messages. Additionally, the platform tracked participants’ daily logins and usage patterns. If a participant did not use the platform for over a week, researchers would contact them by phone to record the reason for their inactivity.

### 2.5. Dietary Management

The health management platform automatically generated personalized dietary plans based on participants’ responses to questionnaire assessments. Participants had the flexibility to adjust these plans according to personal preferences by substituting food items from the platform’s recommendations to better fit their dietary habits. Participants recorded dietary logs for at least two days per week, which the platform analyzed to assess nutritional intake, identify dietary issues, and provide improvement suggestions.

### 2.6. Exercise Management

The platform designed a 12-phase progressive exercise prescription tailored to each participant based on their health status. Participants selected specific exercise activities from the prescribed plan and uploaded detailed information about their daily activities (including type and duration) to the platform. The system calculated the total exercise volume and compared participants’ weekly activity levels with the recommended exercise prescription. These comparisons were used to assess adherence to the plan and provide personalized feedback for improvement.

### 2.7. Clinical Parameter Measurements

Fasting venous blood samples without anticoagulants were collected after an overnight fast of at least 8 h. Fasting blood glucose (FBG) was measured using the glucose oxidase method, while serum lipid levels, including total cholesterol (TC), triglycerides (TG), high-density lipoprotein cholesterol (HDL-C), and low-density lipoprotein cholesterol (LDL-C), were determined enzymatically. All measurements were performed using fully automated biochemical analyzers. Blood pressure was measured using an electronic sphygmomanometer. Participants were asked to sit quietly for at least 5 min before the measurement to ensure relaxation. Blood pressure was measured twice, with an interval of more than 30 s between measurements. The average of the two readings was used for analysis.

### 2.8. Questionnaire Survey

After each health examination, questionnaires were administered to collect demographic information, lifestyle behaviors (e.g., smoking status, alcohol consumption, dietary habits, physical activity level), personal and family medical history, and sleep and psychological conditions. Diabetes status was initially assessed with the following two questions: “Have you been informed by a healthcare professional that you have diabetes?” and “Are you taking glucose-lowering medications?” If the answer was affirmative, the participant was classified as having diabetes. If no prior diagnosis was reported but the participant had a FBG level ≥ 7.0 mmol/L, they were classified as newly diagnosed with diabetes. The diabetes awareness rate was defined as the proportion of individuals who self-reported or were aware of having diabetes among all T2D patients. The diabetes control rate was defined as the proportion of T2D patients at baseline who had controlled blood glucose levels at follow-up. Physical activity levels were classified into low, moderate, and high according to the International Physical Activity Questionnaire (IPAQ) standards [29]. Sleep quality was assessed using the Pittsburgh Sleep Quality Index (PSQI) over the past month [30]. Dietary intake was classified as below, meeting, or exceeding the recommended intake based on the 2016 Chinese Dietary Guidelines [31]. Psychological resilience and emotional disorder risks (e.g., depression, anxiety, loneliness) were assessed using a 7-item scale with a 5-point Likert scale to quantify psychological well-being.

### 2.9. Statistical Analysis

Data analysis was conducted using SPSS version 22.0. Continuous variables were presented as mean ± standard deviation (SD), and categorical variables were presented as frequencies and percentages. Differences between groups were analyzed using Student’s *t*-test or analysis of variance (ANOVA) for continuous variables and Chi-square tests for categorical variables. The significance level was set at *p* < 0.05. For longitudinal analysis, paired *t*-tests or Wilcoxon signed-rank tests were used to evaluate changes in self-management behaviors and clinical parameters over the four-year follow-up period, depending on the normality of the data. Newly diagnosed diabetes patients were classified as well-controlled if their FBG was <7.0 mmol/L at the 2017 follow-up after four years of health management; otherwise, they were considered poorly controlled. Independent sample *t*-tests were used to analyze factors associated with blood glucose control, followed by the establishment of a binary logistic regression model using backward elimination to determine independent factors associated with successful blood glucose control after four years of internet-based health management. Variables included in the regression analysis were those that showed significant associations in univariate analysis. The dependent variable in the model represented blood glucose control status in new-onset T2D patients, with 0 indicating effective control and 1 indicating ineffective control. Multicollinearity among variables was assessed, and the model’s goodness-of-fit was evaluated using the Hosmer–Lemeshow test. The reporting of this study follows the STROBE guidelines [32]. All figures in the results section of the paper were generated using R software (version 4.1.3).

## 3. Results

### 3.1. Basic Characteristics of Eligible Participants in 2013

A total of 30,333 eligible participants were tracked during the 2017 follow-up, of whom 28,026 were non-diabetic and 2307 were diagnosed with T2D at baseline. The results showed significant differences between the non-T2D group and the T2D group in terms of age, gender, and family history of diabetes (*p* < 0.001). Regarding health-related behaviors, the rate of alcohol consumption was significantly higher in the T2D group compared to the non-T2D group (*p* < 0.001). Participants in the non-T2D group smoked and drank less and engaged in more physical activity. Although dietary intake for all participants was generally below recommended levels, the T2D group exhibited more pronounced deficiencies in the intake of various nutritious foods. Most participants had intake levels of milk, nuts, vegetables, and fruits that were far below the recommended standards (Table 1).

### 3.2. Change in Self-Management and Clinical Parameters of Eligible T2D Patients

**Figure 2:**
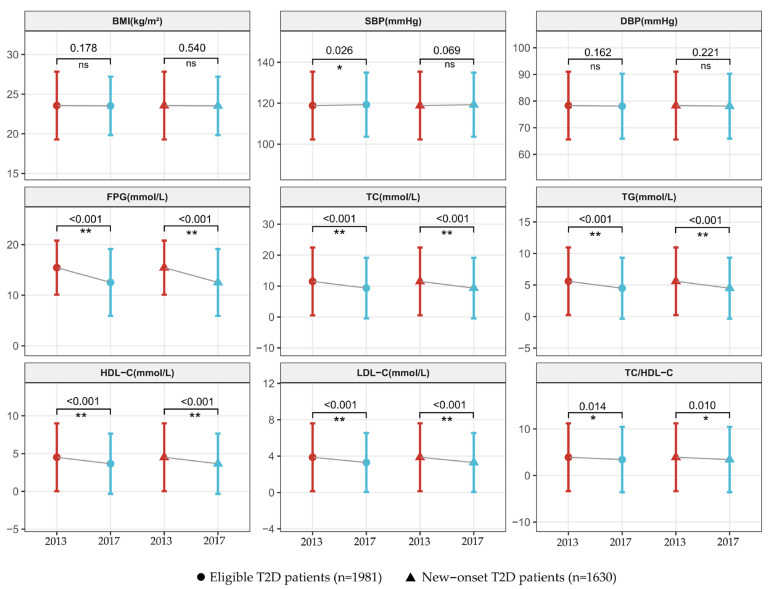
Changes in clinical parameters of eligible T2D patients and new-onset T2D patients after health management. BMI: body mass index; SBP: systolic blood pressure; DBP: diastolic blood pressure; FBG: fasting plasma glucose; TC: total cholesterol; TG: triglycerides; HDL-C: high-density lipoprotein cholesterol; LDL-C: low-density lipoprotein cholesterol. * *p* < 0.05; ** *p* < 0.001; ns: not significant.

After four years of internet-based health management, notable changes were observed in self-management behavior among eligible T2D patients, as summarized in Table 2. The diabetes awareness rate increased from 17.72% in 2013 to 19.84% in 2017, and the diabetes control rate rose significantly from 7.22% to 26.91% over the same period. In terms of self-management behaviors, the smoking cessation rate increased from 5.35% to 7.22% (*p* < 0.001), while the proportion of patients engaging in sufficient exercise rose from 67.49% to 72.19% (*p* < 0.001). Dietary improvements were also observed, with a rise in the intake of fish, eggs, poultry, and livestock (from 46.90% to 48.71%, *p* = 0.005) and a substantial increase in the consumption of soybeans and nuts (from 29.98% to 50.38%, *p* < 0.001). Additionally, sufficient intake of vegetables increased from 20.75% to 22.31% (*p* < 0.001), and fruit intake rose from 10.25% to 11.11% (*p* = 0.024). Regarding clinical parameters, FPG levels showed a significant reduction from 14.16 mmol/L to 11.78 mmol/L (*p* < 0.001). SBP increased slightly from 120.55 mmHg to 121.04 mmHg (*p* = 0.026). Lipid profile measurements also improved, with decreases in TC (*p* < 0.001), TG (*p* < 0.001), and LDL-C (*p* < 0.001). Although HDL-C decreased from 4.06 mmol/L to 3.34 mmol/L (*p* < 0.001), the TC/HDL ratio showed a favorable reduction from 3.85 to 3.48 (Figure 2, Table S1).

### 3.3. Changes in Self-Management and Clinical Parameters of Participants with New-Onset Diabetes

Among the 1630 participants newly diagnosed with diabetes in 2013, significant changes in self-management behaviors and clinical parameters were observed after four years of internet-based health management (Table 2). The smoking cessation rate rose from 3.87% to 5.83% (*p* < 0.001), and the proportion of participants engaging in sufficient exercise increased from 65.28% to 70.61% (*p* < 0.001). Dietary habits also improved, with notable increases in the intake of cereal and potatoes (from 52.27% to 53.68%, *p* = 0.048), fish, eggs, poultry, and livestock (from 46.44% to 48.65%, *p* = 0.002), soybeans and nuts (from 28.65% to 48.22%, *p* < 0.001), vegetables (from 18.65% to 20.18%, *p* = 0.002), and fruits (from 10.43% to 11.66%, *p* = 0.006). For clinical parameters, FPG levels showed a marked reduction from 15.45 to 12.53 mmol/L (*p* < 0.001), underscoring the effectiveness of internet-based health management for new-onset diabetes patients. Additionally, significant decreases were observed in TC, TG, and LDL-C (Figure 2). After four years, the glycemic control rate in these new-onset diabetes patients reached 24.36% (397 participants), demonstrating positive outcomes with sustained internet-based health management.

### 3.4. Factors Related to Blood Glucose Control in Participants with New-Onset Diabetes

After four years of health management, 397 participants (24.36%) achieved well-controlled blood glucose levels, while 1233 participants (75.64%) had poorly controlled blood glucose. As shown in Table 3, female participants demonstrated significantly better blood glucose control than male participants (*p* = 0.003). Participants who adhered to health management guidance on smoking cessation (*p* = 0.002), and increased intake of fish, eggs, poultry, and livestock meat (*p* = 0.001), soybeans and nuts (*p* < 0.001), vegetables (*p* = 0.019), and fruits (*p* < 0.001) were more likely to achieve better blood glucose control. Additionally, improved physical activity, reduced sedentary time, and a positive psychological state were significantly associated with better glycemic control.

### 3.5. Binary Logistic Regression Model of Factors Associated with Blood Glucose Control in New-Onset Patients

**Figure 3:**
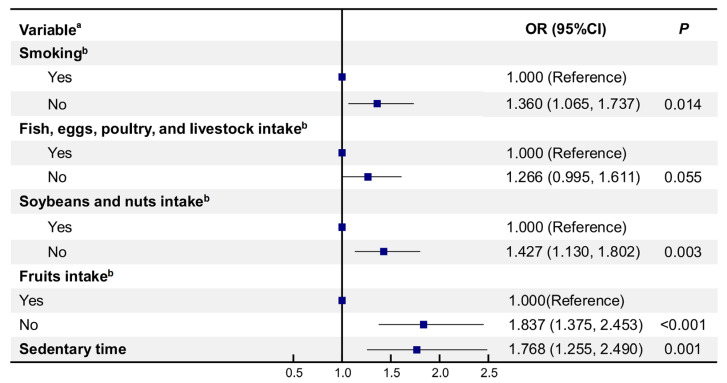
Binary logistics model of factors related to blood glucose control after health management in participants with new-onset diabetes. ^a^ The dependent variable was blood glucose control, assigned the values: effective blood glucose control = 0, ineffective blood glucose control = 1. ^b^ “Yes” indicates that health-related behavior improved after following health management guidance, whereas “No” indicates no change in behavior.

A binary logistic regression analysis was conducted to identify factors associated with blood glucose control in participants with new-onset diabetes after health management (Figure 3). Smoking cessation was significantly associated with better blood glucose control, with participants who quit smoking having a lower risk of poor outcomes compared to those who continued smoking (OR = 1.360, 95% CI: 1.065–1.737). Dietary intake of soybeans and nuts increased the likelihood of effective blood glucose control by 42.7% (OR = 1.427, 95% CI: 1.130–1.802), while increased fruit intake was associated with an 83.7% higher likelihood of good control (OR = 1.837, 95% CI: 1.375–2.453). Reducing sedentary time also improved blood glucose control, with a 76.8% greater likelihood of effective control (OR = 1.768, 95% CI: 1.255–2.490). Overall, reducing tobacco use, increasing intake of soybeans, nuts, and fruits, and decreasing sedentary time were key factors associated with better blood glucose control in patients with new-onset diabetes.

## 4. Discussion

This study demonstrates that internet-based health management can significantly improve blood glucose control, clinical parameters, and health-related behaviors in T2D patients. After four years of health management, the average FBG level in T2D patients significantly decreased, and the blood glucose control rate increased substantially. Additionally, lipid levels improved, including reductions in TC, TG, and LDL-C, further confirming the effectiveness of internet-based health management. These results emphasize the role of internet health platforms in improving metabolic health.

Based on an 18-month longitudinal study, Chen et al. found that using an online diabetes self-management system for telemedicine led to significant improvements in healthy eating, medication adherence, and average HbA1c levels among T2D patients [33]. Park et al. found that interventions based on mobile health applications significantly improved blood glucose control rates and health behaviors in T2D patients [34]. It is important to note that, in addition to statistical significance, the actual clinical impact of mobile health interventions on patient health should also be considered. Ruiz-Leon et al. reported that after 12 weeks of mobile health behavioral treatment for T2D, the intervention group showed significant reductions in SBP (−4.5 mmHg), DBP (−2.4 mmHg), weight (−0.8 kg), BMI (−0.3 kg/m^2^), waist circumference (−1.0 cm), and TG levels (−20.0 mg/dL) [35]. Moreover, remote health management has been shown to effectively reduce acute complications in T2D patients and improve quality of life, further supporting the effectiveness of internet health management in diabetes care. Sjöblom et al. reported that a 12-week smartphone-based dietary education program led to greater reductions in the daily intake of saturated fats (β = −4.1, 95% CI: −7.9, −0.2) and unsaturated fats (β = −6.9, 95% CI: −13.5, −0.4) among T2D patients compared to controls, along with lower TG levels (β = −0.33, 95% CI: −0.60, −0.05) [36].

Through the internet health management platform, participants showed significant improvements in health-related behaviors, particularly in areas such as diet, physical activity, and a reduction in unhealthy habits. Notably, smoking cessation, increased intake of soybeans, nuts, and fruits, as well as decreased sedentary behavior, were strongly associated with better blood glucose control. Smoking cessation plays a key role in diabetes management, as smoking impairs pancreatic β-cell function and exacerbates insulin resistance [37,38]. Our findings confirmed that T2D patients who quit smoking showed significant improvements in blood glucose control, consistent with the evidence presented by Burgoa, who emphasized the importance of smoking cessation in chronic disease management [39].

Moreover, the increased intake of soybeans and nuts was associated with improved blood glucose control, likely due to their high content of unsaturated fatty acids and antioxidants [40,41,42]. Similarly, a higher consumption of fresh fruits, rich in fiber and polyphenols, was linked to better blood glucose control. This effect may be attributed to the role of fruits in slowing glucose absorption and enhancing insulin sensitivity [43,44]. These dietary changes highlight the importance of incorporating nutritional guidance into diabetes health management plans. Xu et al. found that, compared with the control group at the 6-month mark, after the intervention group for T2D received the management based on the combination of mobile devices and behavioral theory, the intake of energy, fat, and carbohydrates in this group significantly decreased, and at the same time, the moderate-intensity physical activity significantly increased [45]. Increased physical activity, another key aspect of the health management program, was shown to improve insulin sensitivity, thereby facilitating glucose uptake and utilization [46].

Additionally, Chen et al. found that remote self-management of telemedicine significantly improved positive attitudes, healthy coping mechanisms, and problem-solving behaviors in T2D patients [33]. Recent studies have shown that mobile health interventions have significant effects on improving patients’ mental health, cognitive flexibility, distress, sleep quality, and demonstrate good efficacy and acceptability [47]. Although mobile health is a promising tool for psychological care, in this study, there was no significant change in sleep quality among T2D patients after health management, and differences in sleep quality did not significantly impact blood glucose control. However, participants with better psychological states had a higher proportion of good blood glucose control (29.47%), while those with poorer psychological states had a higher proportion of poor blood glucose control (76.81%). This suggests that mental health may have a certain impact on blood glucose control, potentially through reducing stress hormone secretion (such as cortisol), which positively influences glucose metabolism [48,49]. Although the specific duration of platform usage was not recorded in this study, previous research has indicated that nighttime screen exposure can disrupt circadian rhythms by inhibiting melatonin secretion, thereby affecting sleep quality [50]. Therefore, future studies should consider the reasonable use of platform time to balance the metabolic benefits of internet-based health management with the maintenance of sleep health.

Internet-based health management provides potential insights into cost-effective methods for diabetes care, offering a convenient and sustainable alternative that could be particularly beneficial in developing countries and regions. Unlike traditional face-to-face consultations, internet platforms eliminate geographical and temporal constraints, enabling patients to receive continuous health support [51]. The results of this study demonstrate that personalized feedback and ongoing monitoring through internet platforms not only enhance patients’ self-management and adherence but also lead to improved clinical outcomes. These findings suggest that remote health interventions can be as effective as traditional face-to-face care in managing diabetes, reducing acute complications, and improving quality of life [52].

### 4.1. Strengths and Limitations

The advantages of this study include its large sample size, relatively long follow-up period, and comprehensive assessment of individual health behaviors through an internet platform. Over the past decade, despite the rapid advancement of continuous glucose monitoring systems (CGM) and novel medications, our study explored how to utilize the most direct and easily accessible internet-based methods to overcome geographical barriers, providing personalized health management solutions for diabetes patients in resource-limited areas. This approach offers potential insights for diabetes management in developing countries and regions.

However, this study also has several limitations. First, the study targeted employees of large enterprises, and participants may have dropped out during the long follow-up period due to various factors (e.g., job changes), resulting in a relatively high attrition rate. Second, self-reported dietary information and physical activity data may introduce bias, affecting the accuracy of dietary intake and physical activity assessments. Third, the rapid advancements in diabetes management tools may lead to differences between the intervention measures in this study and current technological standards in terms of effectiveness. Additionally, FBG as an indicator of blood glucose control has limitations, as it cannot fully reflect blood glucose fluctuations or long-term blood glucose levels. Nevertheless, due to resource and time constraints, we were unable to regularly measure HbA1c levels or collect patients’ medication information, which may have impacted on the comparability and applicability of the results. Finally, while the longitudinal design of this study allows for the reflection of long-term changes in health behaviors, it is subject to confounding factors such as differences in diabetes duration, baseline health status, and socioeconomic status, which may affect the broad applicability of the results.

### 4.2. The New Directions for Future Research

Future research should address the limitations identified in this study. Firstly, medication use data should be incorporated, and the interaction between medication adherence and behavioral interventions should be analyzed to provide a more comprehensive assessment of the intervention’s effects. Secondly, more proactive incentives and patient education should be implemented, along with further optimization of the health management platform, to reduce attrition rates and improve adherence. Additionally, integrating new diabetes management technologies [53], such as CGM and wearable devices, along with more precise indicators like HbA1c levels, can enhance blood glucose control and the assessment of physical activity. Furthermore, future studies should examine the potential impact of disease duration and other factors on intervention outcomes and adopt standardized dietary assessment methods to minimize confounding factors affecting the results.

## 5. Conclusions

Internet-based health management can effectively improve blood glucose control and health behaviors in T2D patients. Key lifestyle changes, such as increased physical activity, improved dietary habits, and smoking cessation, are crucial factors influencing blood glucose control in T2D patients. The study demonstrates that even in resource-limited environments, internet platforms, as a cost-effective and accessible option, can overcome geographical barriers to help T2D patients achieve long-term and effective disease management, providing a reference for diabetes management in developing countries and regions.

## Figures and Tables

**Table 1:** Demographic and health characteristics of eligible participants in 2013 (n = 30,333).

Characteristic	Total (n = 30,333)	Non-T2DM (n = 28,026)	T2DM (n = 2307)	*p*
Age, mean ± SD	35.71 ± 8.17	35.64 ± 8.08)	36.61 ± 9.15	<0.001
Gender, n (%)				<0.001
Male	19,236 (63.42)	17,558 (62.65)	1678 (72.74)	
Female	11,097 (36.58)	10,468 (37.35)	629 (27.26)	
Family history of diabetes (father), n (%)	1368 (4.51)	1200 (4.28)	168 (7.28)	<0.001
Family history of diabetes (mother), n (%)	1080 (3.56)	953 (3.40)	127 (5.50)	<0.001
Tobacco use, n (%)				<0.001
Non-smoking	19,358 (63.85)	18,105 (64.64)	1253 (54.31)	
Current smoking	10,075 (33.23)	9143 (32.64)	932 (40.40)	
Quit smoking	885 (2.92)	763 (2.72)	122 (5.29)	
Alcohol consumption, n (%)				0.001
No alcoholic beverages	18,635 (65.45)	17,215 (65.72)	1420 (62.42)	
Drinking	9835 (34.55)	8980 (34.28)	855 (37.58)	
Cereals and potato intake, n (%)				0.339
Below	13,119 (43.25)	12,077 (43.09)	1042 (45.17)	
Moderate	8675 (28.60)	8067 (28.78)	608 (26.35)	
Higher	8539 (28.15)	7882 (28.12)	657 (28.48)	
Fish, eggs, poultry, and livestock meat intake, n (%)				0.011
Below	15,246 (50.26)	14,030 (50.06)	1216 (52.71)	
Moderate	7600 (25.06)	7039 (25.12)	561 (24.32)	
Higher	7487 (24.68)	6957 (24.82)	530 (22.97)	
Milk and dairy products intake, n (%)				0.002
Below	26,839 (88.48)	24,844 (88.65)	1995 (86.48)	
Moderate	3494 (11.52)	3182 (11.35)	312 (13.52)	
Soybeans and nuts intake, n (%)				<0.001
Below	20,058 (66.13)	18,422 (65.73)	1636 (70.91)	
Moderate	1793 (5.91)	1699 (6.06)	94 (4.07)	
Higher	8482 (27.96)	7905 (28.21)	577 (25.01)	
Vegetables intake, n (%)				<0.001
Below	22,798 (75.16)	20,975 (74.84)	1823 (79.02)	
Moderate	5180 (17.08)	4851 (17.31)	329 (14.26)	
Higher	2355 (7.76)	2200 (7.85)	155 (6.72)	
Fruits intake, n (%)				0.002
Below	26,322 (86.78)	24,258 (86.56)	2064 (89.47)	
Moderate	2994 (9.87)	2826 (10.08)	168 (7.28)	
Higher	1017 (3.35)	942 (3.36)	75 (3.25)	
Quality of sleep, n (%)				<0.001
Very good	6922 (22.84)	6447 (23.03)	475 (20.59)	
Fair	18,410 (60.75)	17,133 (61.19)	1277 (55.35)	
Not good	4066 (13.42)	3644 (13.01)	422 (18.29)	
Very bad	908 (3.00)	775 (2.77)	133 (5.77)	
Physical activity, n (%)				0.011
Low	8983 (29.61)	8213 (29.30)	770 (33.38)	
Medium	13,771 (45.40)	12,811 (45.71)	960 (41.61)	
High	7579 (24.99)	7002 (24.98)	577 (25.01)	
Sleeping time, mean ± SD	7.23 ± 1.18	7.23 ± 1.17	7.12 ± 1.33	<0.001
Psychological score, mean ± SD	17.87 ± 5.10	17.80 ± 5.08	18.63 ± 5.32	<0.001
Sedentary time, mean ± SD	5.11 ± 2.78	5.12 ± 2.75	4.98 ± 3.10	0.018

Numerical variables are expressed as mean ± standard deviation (SD), and categorical variables are expressed as frequency (%). Non-T2D: non-type 2 diabetes group; T2D: type 2 diabetes group. Some variables contain missing data, and percentages are calculated based on valid responses.

**Table 2:** Health behavior and psychological profile changes in eligible and new-onset T2D patients before vs. after health management.

Parameter	Eligible T2D Patients (n = 1981)		*p*	New-Onset T2D Patients (n = 1630)		*p*
	2013	2017		2013	2017	
Health behaviors						
Quit smoking, n (%)	106 (5.35)	143 (7.22)	<0.001	63 (3.87)	95 (5.83)	<0.001
Sufficient exercise, n (%)	1337 (67.49)	1430 (72.19)	<0.001	1064 (65.28)	1151 (70.61)	<0.001
Sufficient cereal and potato intake, n (%)	1094 (55.22)	1116 (56.34)	0.071	852 (52.27)	875 (53.68)	0.048
Sufficient fish, eggs, poultry, and livestock meat intake, n (%)	929 (46.90)	965 (48.71)	0.005	757 (46.44)	793 (48.65)	0.002
Sufficient milk and dairy intake, n (%)	272 (13.73)	271 (13.68)	0.904	236 (14.48)	235 (14.42)	0.898
Sufficient soybeans and nuts intake, n (%)	594 (29.98)	998 (50.38)	<0.001	467 (28.65)	786 (48.22)	<0.001
Sufficient vegetables intake, n (%)	411 (20.75)	442 (22.31)	<0.001	304 (18.65)	329 (20.18)	0.002
Sufficient fruits intake, n (%)	203 (10.25)	220 (11.11)	0.024	170 (10.43)	190 (11.66)	0.006
Times of drinking per week, mean ± SD	0.96 ± 2.05	1.00 ± 2.11	0.533	0.50 ± 1.53	0.50 ± 1.46	0.932
Sleep and psychological condition						
Quality of sleep, n (%)			0.140			0.090
Very good	410 (20.73)	346 (17.50)		349 (21.44)	290 (17.82)	
Fair	1082 (54.66)	1098 (55.45)		883 (54.17)	897 (55.06)	
Not good	373 (18.84)	428 (21.65)		300 (18.42)	350 (21.52)	
Very bad	114 (5.77)	106 (5.40)		97 (5.97)	91.28 (5.60)	
Sleeping time, mean ± SD	7.13 ± 1.33	7.07 ± 1.25	1.162	7.15 ± 1.34	7.09 ± 1.25	0.209
Psychological score, mean ± SD	18.72 ± 5.31	18.69 ± 5.19	0.833	18.80 ± 5.29	18.8 ± 5.16	0.993

**Table 3:** Analysis of factors influencing blood glucose control in participants with new-onset diabetes (n = 1630).

Factors	Well Controlled (n = 397)	Poorly Controlled (n = 1233)	*p*
Age (years), mean ± SD	37.97 ± 7.84	37.91 ± 8.45	0.890
Gender, n (%)			0.003
Male	245 (22.17)	860 (77.83)	
Female	152 (28.95)	373 (71.05)	
Family history of diabetes (father), n (%)			0.535
Yes	30 (27.79)	82 (72.21)	
No	367 (24.18)	1151 (75.82)	
Family history of diabetes (mother), n (%)			0.447
Yes	18 (20.93)	68 (79.07)	
No	379 (24.55)	1165 (75.45)	
** Changes in behavioral factors according to the guidance of health management, n (%) **			
Smoking			0.002
Yes	270 (26.95)	732 (73.05)	
No	127 (20.22)	501 (79.78)	
Drinking			0.964
Yes	250 (24.32)	778 (75.68)	
No	147 (24.42)	455 (75.58)	
Cereals and potato intake			0.231
Yes	167 (25.93)	477 (74.07)	
No	230 (23.33)	756 (76.67)	
Fish, eggs, poultry, and livestock meat intake			0.001
Yes	169 (28.89)	416 (71.11)	
No	228 (21.82)	817 (78.18)	
Milk and dairy products intake			0.731
Yes	84 (25.07)	251 (74.93)	
No	313 (24.17)	982 (75.83)	
Soybeans and nuts intake			<0.001
Yes	216 (28.99)	529 (71.01)	
No	181 (20.45)	704 (79.55)	
Vegetables intake			0.019
Yes	103 (29.10)	251 (70.90)	
No	294 (23.04)	982 (76.96)	
Fruits intake			<0.001
Yes	101 (37.69)	167 (62.31)	
No	296 (21.73)	1066 (78.27)	
Physical activity			0.037
Yes	242 (26.30)	678 (73.70)	
No	155 (21.83)	555 (78.17)	
Sedentary time			<0.001
Yes	63 (37.06)	107 (62.94)	
No	334 (22.88)	1126 (77.12)	
Sleeping time			0.698
Yes	253 (24.05)	799 (75.95)	
No	144 (24.91)	434 (75.09)	
Sleeping quality			0.790
Yes	89 (23.67)	287 (76.33)	
No	308 (24.56)	946 (75.44)	
Psychological state			0.022
Yes	89 (29.47)	213 (70.53)	
No	308 (23.19)	1020 (76.81)	

## Data Availability

The raw data supporting the conclusions of this article will be made available by the authors on request.

## Supplementary Materials

The following supporting information can be downloaded at: https://www.mdpi.com/article/10.3390/healthcare13050553/s1, Table S1: Changes of clinical parameters of eligible and new-onset T2D patients.
