# Supplementary material for: Influence of Internet-Based Health Management on Control of Blood Glucose in Patients with Type 2 Diabetes: A Four-Year Longitudinal Study
Source: Healthcare (Basel). 2025 Mar 4;13(5):553. doi: 10.3390/healthcare13050553 (PMC11899629; doi:10.3390/healthcare13050553)
Supplement: Supplementary file 1 [file healthcare-13-00553-s001.zip › healthcare-3484895-supplementary.pdf]

Table S1. Changes of clinical parameters of eligible and new-onset T2D patients.

**Clinical parameters**	**Eligible T2D patients (n=1981)**		***P***	**New-onset T2D patients (n = 1630)**		***P***
	**2013**	**2017**		**2013**	**2017**	
BMI(kg/m^2)	23.89 ± 4.19	23.81 ± 3.66	.178	23.56 ± 4.28	23.52 ± 3.69	.540
SBP(mmHg)	120.55 ± 16.88	121.04 ± 16.27	.026	118.83 ± 16.58	119.27 ± 15.66	.069
DBP(mmHg)	79.31 ± 12.79	79.08 ± 12.30	.162	78.30 ± 12.73	78.08 ± 12.19	.221
FPG(mmol/L)	14.16 ± 5.75	11.78 ± 6.36	<.001	15.45 ± 5.34	12.53 ± 6.61	<.001
TC(mmol/L)	10.34 ± 10.34	8.58 ± 9.12	<.001	11.49 ± 10.97	9.35 ± 9.78	<.001
TG(mmol/L)	5.11 ± 5.07	4.21 ± 4.56	<.001	5.60 ± 5.36	4.49 ± 4.84	<.001
HDL-C(mmol/L)	4.06 ± 4.24	3.34 ± 3.74	<.001	4.51 ± 4.49	3.66 ± 3.99	<.001
LDL-C(mmol/L)	3.52 ± 3.49	3.08 ± 3.02	<.001	3.87 ± 3.73	3.30 ± 3.24	<.001
TC/HDL-C	3.85 ± 7.09	3.48 ± 6.87	.014	3.92 ± 7.29	3.44 ± 7.03	.010

BMI: body mass index; SBP: systolic blood pressure; DBP: diastolic blood pressure; FBG: fasting plasma glucose; TC: total cholesterol; TG: triglycerides; HDL-C: high-density lipoprotein cholesterol; LDL-C: low-density lipoprotein cholesterol.
